# In Situ Fabrication of BiOCl@Bi_2_S_3_@ZnIn_2_S_4_ Double Z-Scheme Heterojunctions for Enhanced Photocatalytic Degradation Performance

**DOI:** 10.3390/molecules31162843

**Published:** 2026-08-14

**Authors:** Ligang Ma, Tingting Chen, Jingxuan Zhou, Jiulei Zhao, Xinlan Li, Huilin Jiang, Liping Li, Xiaoqian Ai

**Affiliations:** 1 School of Electronic Engineering, Nanjing Xiaozhuang University, Nanjing 211171, China; lgma@njxzc.edu.cn (L.M.);; 2 School of Physics and Electronic Information, Jiangsu Second Normal University, Nanjing 210013, China

**Keywords:** BiOCl, ZnIn_2_S_4_, heterojunction, photocatalysis, in situ synthesis

## Abstract

Organic pollutants in industrial wastewater present a severe threat to both the environment and human health. Photocatalytic technology, recognized for its eco-friendliness and high efficiency, has become a leading approach for degrading such pollutants. In this work, BiOCl nanosheets were first synthesized using a hydrothermal method. Subsequently, an anion exchange reaction with TAA in an oil bath generated a Bi_2_S_3_ intermediate layer on the BiOCl surface, followed by the in situ growth of ZIS nanostructures, successfully constructing a BiOCl@Bi_2_S_3_@ZIS double Z-scheme heterojunction. By adjusting the amount of BiOCl, the interface contact and dispersion of the heterojunction were optimized. Characterization results demonstrate that the BiOCl@ZIS-25 heterojunction possesses the highest specific surface area (103.5 m^2^·g^−1^) and the most efficient charge separation. Under visible light irradiation, it achieved 97.88% degradation of methylene blue within 20 min, with a reaction rate constant 8 and 4 times higher than those of pure BiOCl and ZIS, respectively. Mechanistic investigations indicate that Bi_2_S_3_ interlayer acts as an electron-transfer bridge between BiOCl and ZIS, establishing a double Z-scheme charge transfer pathway that significantly enhanced the separation and utilization efficiency of photogenerated charge carriers. This study offers valuable insights for designing highly efficient and stable photocatalytic composite materials.

## 1. Introduction

The rapid advancement of modern industry, while driving social progress, has also imposed profound and multifaceted environmental burdens. One of the most pressing issues is the discharge of industrial wastewater, which typically contains substantial amounts of organic pollutants [1,2]. Untreated release of such wastewater not only reduces dissolved oxygen levels in aquatic systems but also introduces toxic compounds that can severely disrupt ecosystems and ultimately endanger human health [3]. Consequently, researchers have been actively pursuing environmentally benign and effective strategies to degrade these hazardous organic contaminants [4,5,6]. Among various remediation technologies, photocatalysis has gained prominence due to its operational simplicity, eco-compatibility, and high degradation efficiency [7,8,9,10]. Under light irradiation, electrons in the valence band of a photocatalyst are excited to the conduction band, generating strongly reductive electrons in the conduction band and leaving highly oxidative holes in the valence band. These photogenerated electrons and holes can subsequently interact with organic dye molecules, producing reactive free radicals that mineralize the dyes into harmless products such as water and carbon dioxide [11,12,13].

To date, a variety of low-cost photocatalysts have been developed, including metal oxides [6,14,15], sulfides [16,17,18], bismuth-based compounds [19,20,21], inorganic carbon nitrides [22,23], and organic materials [24,25]. Among them, ternary metal sulfide ZnIn_2_S_4_ (ZIS) [26,27,28,29,30,31] has attracted considerable attention for its suitable band structure, non-toxicity, facile synthesis, and good stability—especially in sunlight-driven photocatalytic degradation of organic pollutants. Despite these advantages, pure ZnIn_2_S_4_ tend to undergo nanoparticle aggregation during preparation due to the lack of fixed nucleation centers, which reduces the specific surface area of the photocatalyst [32,33]. Moreover, the rapid recombination of photogenerated electrons and holes in single-phase ZIS leads to relatively low photocatalytic degradation efficiency. To mitigate nanoparticle aggregation and promote charge-carrier separation, several strategies have been explored, such as elemental doping [34], morphology control [35], and constructing heterojunctions with other semiconductors or organic ligands [36,37,38,39]. In particular, coupling two semiconductors with staggered band structures to form a heterojunction is an effective approach. This not only broadens the light-harvesting range but also helps suppress particle aggregation during fabrication. More importantly, the built-in interface facilitates the separation of photogenerated charge carriers, thereby boosting photocatalytic performance. Hence, identifying suitable semiconductors that can form well-matched heterojunctions with ZIS is of great research interest.

In recent years, bismuth oxychloride (BiOCl) has drawn attention owing to its good crystallinity, appropriate band structure, and excellent stability. As a wide-band-gap semiconductor (~3.5 eV), BiOCl possesses a distinctive layered structure composed of [Cl–Bi–O–Bi–Cl] sheets stacked along the c-axis via non-bonding interactions between chlorine atoms [40,41,42]. For instance, Jiang et al. [43] fabricated a ZIS@BiOCl heterojunction by growing BiOCl nanosheets on ZIS, which significantly enhanced photocatalytic degradation efficiency. Similarly, Zou et al. [40] synthesized ZIS via a hydrothermal route and then deposited BiOCl nanoparticles via a precipitation method; the intermediate formation of Bi_2_S_3_ eventually yielded a BiOCl/Bi_2_S_3_/ZIS double Z-scheme heterojunction with further improved activity.

In this work, BiOCl nanosheets were first prepared by a hydrothermal method, followed by the in situ growth of ZIS nanostructures on their surface using an oil-bath approach. Notably, during the oil-bath step, sulfur ions released from the decomposition of thioacetamide preferentially replaced surface chloride ions on BiOCl, leading to the in situ formation of a Bi_2_S_3_/BiOCl hetero-interface. Subsequently, ZIS nanostructures were grown in situ on this intermediate layer, resulting in a BiOCl@Bi_2_S_3_@ZIS double Z-scheme heterojunction through a simple and low-cost route. The amount of BiOCl was varied to optimize the interfacial contact and dispersion uniformity of the heterojunction. The microstructure and optical properties of the as-prepared BiOCl@ZIS heterojunctions were systematically characterized. Finally, the photocatalytic performance was evaluated by monitoring the degradation of methylene blue dye under visible-light irradiation.

## 2. Results and Discussion

**Figure 1:**
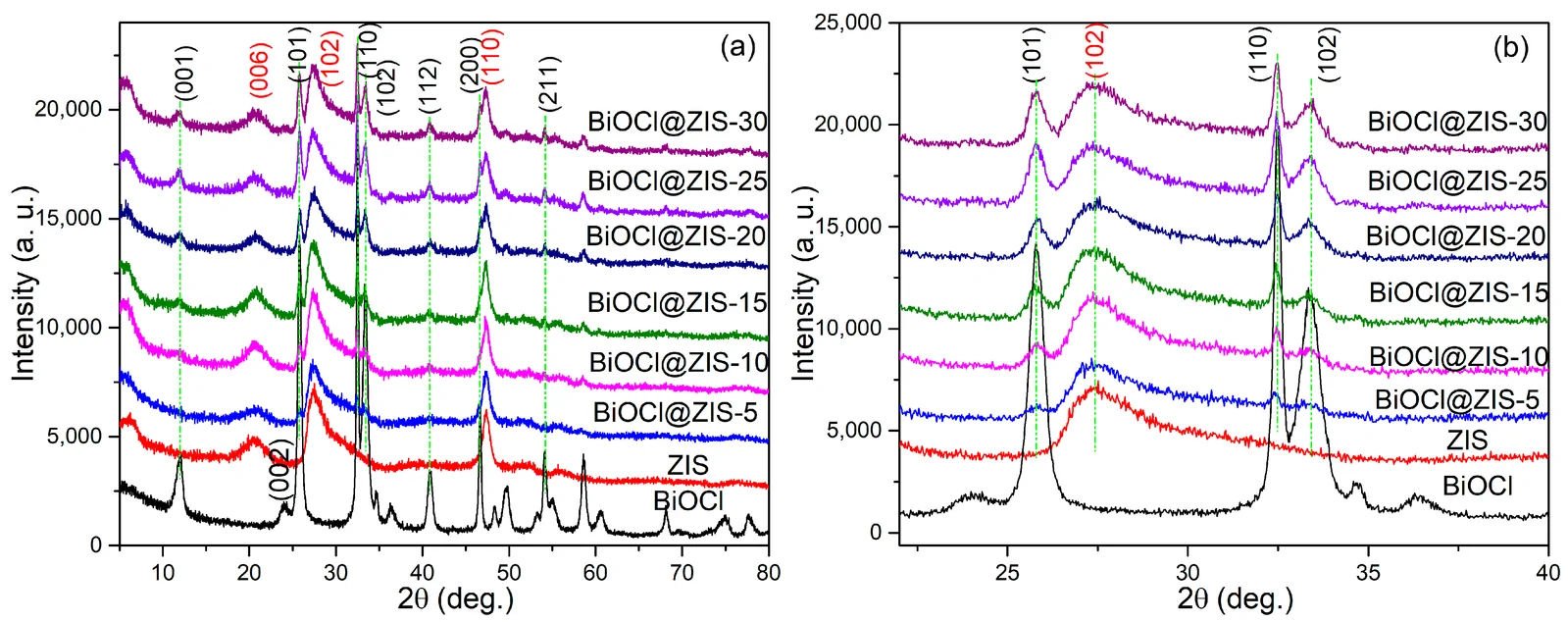
(**a**) XRD patterns of BiOCl, ZIS, and BiOCl@ZIS heterojunction, (**b**) magnified view of XRD patterns.

Figure 1 illustrates the XRD patterns and the corresponding magnified views of the characteristic diffraction peaks for various samples.

**Figure 2:**
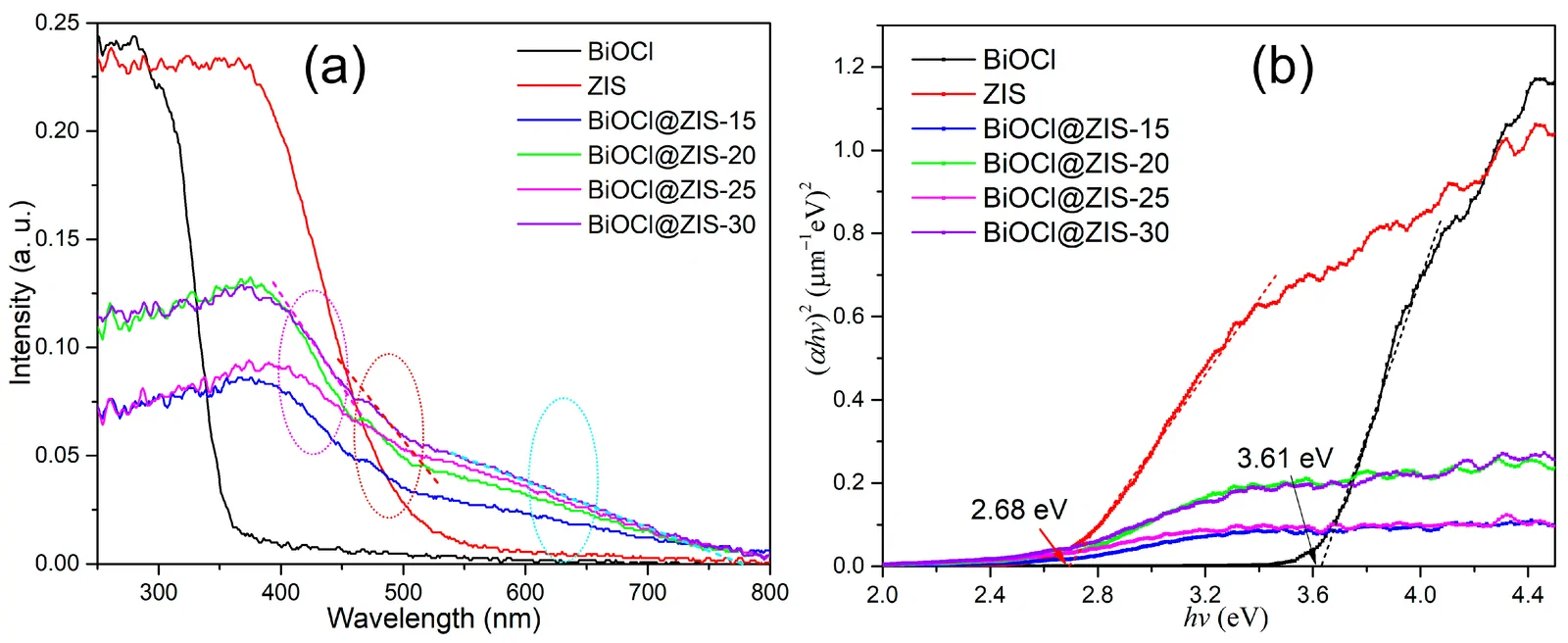
(**a**) UV-vis diffuse reflectance spectra and (**b**) corresponding Tauc plots of BiOCl, ZIS, and BiOCl@ZIS heterojunction.

The BiOCl nanosheets, prepared via a hydrothermal method, exhibit distinct diffraction peaks at 11.9°, 23.8°, 25.8°, 32.5°, 33.3°, 34.6°, 36.3°, 40.7°, 46.6°, 49.7°, 54.4° and 58.6°, which perfectly match the (001), (002), (101), (110), (102), (111), (003), (112), (200), (211) and (212) crystal planes of tetragonal BiOCl (JCPDS No. 06-0249) [44,45], confirming high crystallinity and phase purity. The crystal planes of the main diffraction peaks have been listed, as shown in black in Figure 1. For pure ZIS, characteristic peaks at 20.6°, 27.3° and 47.4° correspond to the (006), (102) and (110) planes of the hexagonal wurtzite structure (JCPDS No.65-2023) [16,17], as shown in the red parentheses in Figure 1. Magnified XRD patterns reveal that as the BiOCl content increases, diffraction signals of BiOCl gradually emerge and intensify alongside the ZIS peaks. This confirms the successful formation of BiOCl@ZIS composites with compositions consistent with the experimental design. Notably, the coexistence of the BiOCl (200) plane and the hexagonal ZIS (110) plane suggests that the heterojunction forms at the interface between the layered ZIS and BiOCl nanosheets. This intimate contact is conducive to enlarging the contact area, thereby enhancing the photoelectric response and charge transport capability [44,46]. Furthermore, the partial overlap of BiOCl (002) and (211) peaks with the (101) and (501) reflections of orthorhombic Bi_2_S_3_ hints at the potential formation of a Bi_2_S_3_ byproduct. This phase likely originates from the in situ substitution of surface chloride ions by sulfide ions during the growth process, a hypothesis further supported by UV-vis absorption spectroscopy in Figure 2.

The light-harvesting properties and optical bandgaps were investigated using UV-vis diffuse reflectance spectroscopy. As shown in Figure 2, pure BiOCl and ZIS exhibit absorption edges at approximately 350 nm and 500 nm, respectively. In contrast, the BiOCl@ZIS heterojunction displays three distinct absorption edges (highlighted by the dashed box in Figure 2a). Based on literature, black Bi_2_S_3_ typically presents an absorption edge near 800 nm [46,47]; thus, these three edges are attributed to Bi_2_S_3_, ZIS, and BiOCl. The physical appearance (Figure S1 in Supporting Information) shows white BiOCl and yellow ZIS. While a physical mixture would appear light yellow, the in situ-grown BiOCl@ZIS heterojunction appears brown/dark, indicating that TAA reacts with the BiOCl surface to form a Bi_2_S_3_ intermediate phase. This transition layer is expected to bridge the energy levels and promote charge separation. Tauc plots (Figure 2b) estimate the optical bandgaps of BiOCl and ZIS to be approximately 3.61 eV and 2.68 eV, respectively.

**Figure 3:**
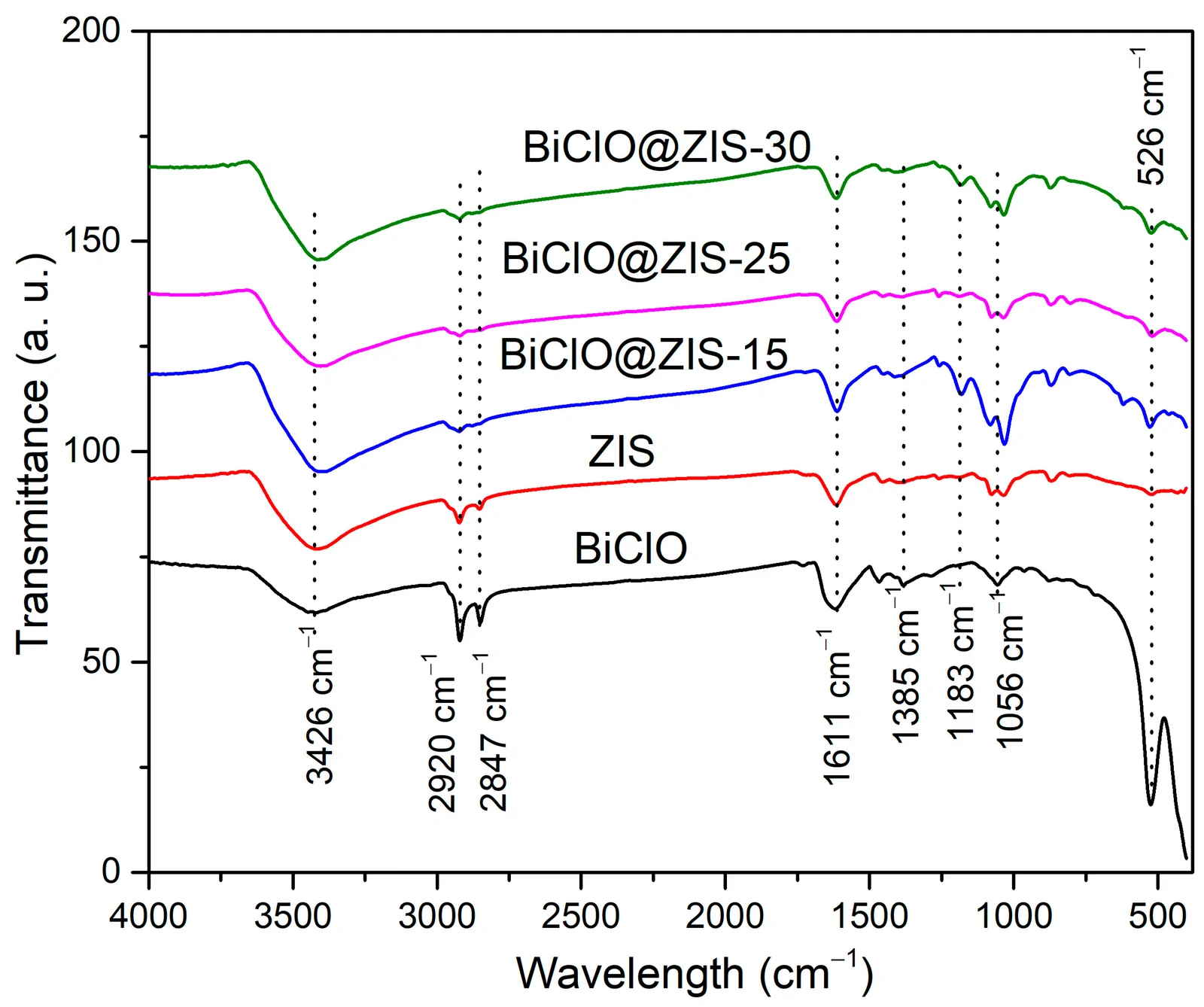
FTIR spectroscopy of BiOCl, ZIS, and BiOCl@ZIS heterojunction.

Figure 3 displays the FTIR spectra used to identify functional groups. All samples show exhibit distinct absorption peaks at 3426 cm^−1^ and 1611 cm^−1^, assigned to the O-H stretching vibration of water molecules and the C=C stretching vibration, respectively [48,49]. Additionally, weaker signals near 2920 cm^−1^ and 2847 cm^−1^ can be observed, which are attributed to residual surface -CH_2_ groups [50]. For pure BiOCl, the sharp peaks at 1385 cm^−1^ and 526 cm^−1^ [40,51], which can be ascribed to the Bi–Cl and Bi–O bending vibrations. It is noteworthy that the Bi–O bending vibration peak at 526 cm^−1^ is also clearly visible in the BiOCl@ZIS heterojunction, further confirming the successful construction of a heterojunction structure between ZIS and BiOCl.

**Figure 4:**
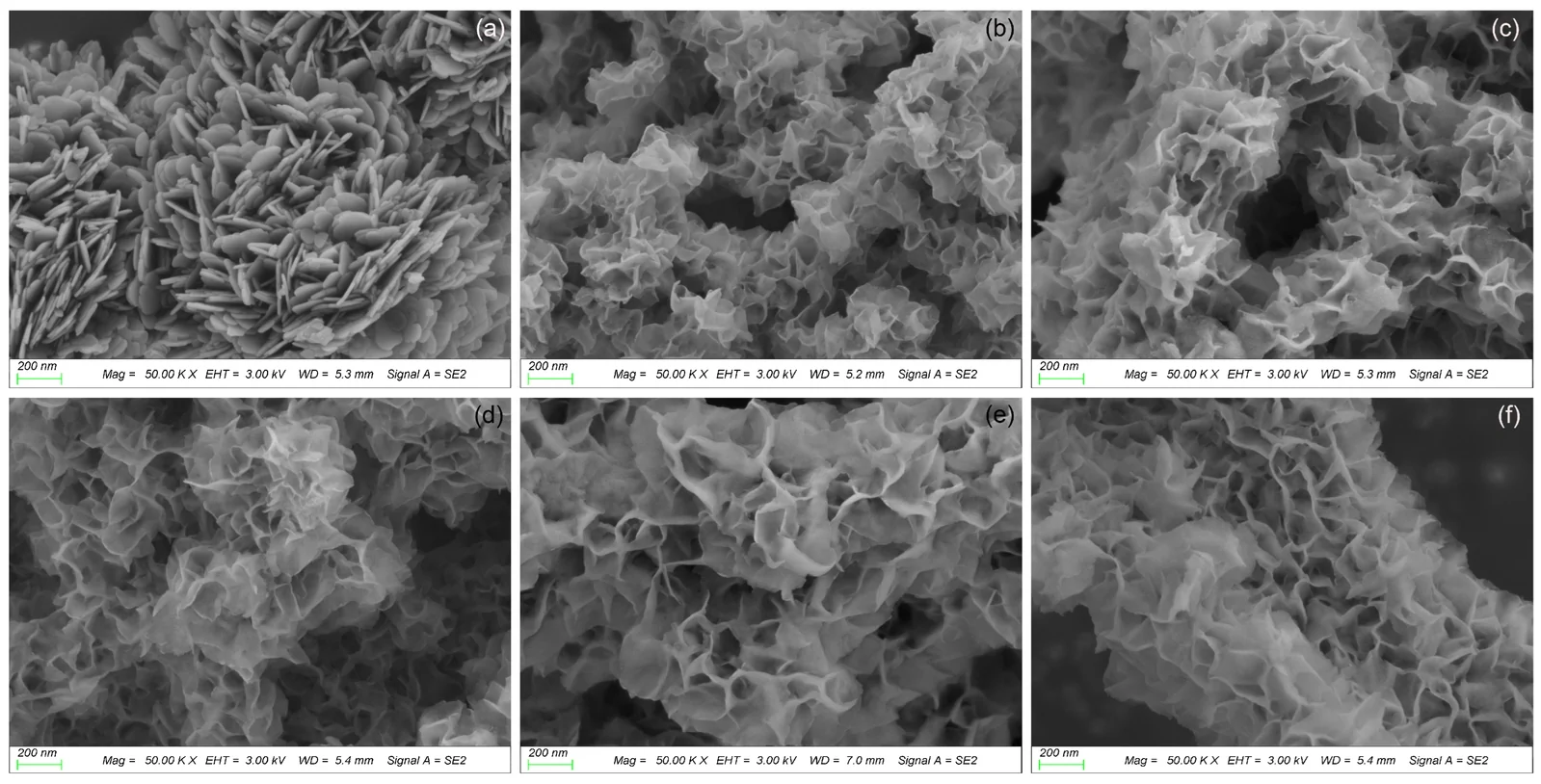
SEM images of (**a**) BiOCl, (**b**) ZIS, (**c**) BiOCl@ZIS-5, (**d**) BiOCl@ZIS-15, (**e**) BiOCl@ZIS-25, and (**f**) BiOCl@ZIS-30 heterojunction.

Figure 4 presents the SEM morphologies of the BiOCl, ZIS, and BiOCl@ZIS composites. As shown, BiOCl displays a thin nanosheet structure, while ZIS exhibits a three-dimensional nanoflower-like morphology assembled from nanosheets. Comparing the two reveals that the nanosheets of BiOCl are thicker than those of ZIS. In the BiOCl@ZIS heterojunction, the morphology is mainly composed of nanosheets, most of which consist of BiOCl and ZIS nanosheets. Additionally, certain regions show morphological features that differ from both ZIS and BiOCl, which are attributed to the anion exchange between BiOCl and TAA leading to the formation of Bi_2_S_3_ nanostructures. To further investigate the microstructure of BiOCl and ZIS, TEM characterization was performed on the samples.

**Figure 5:**
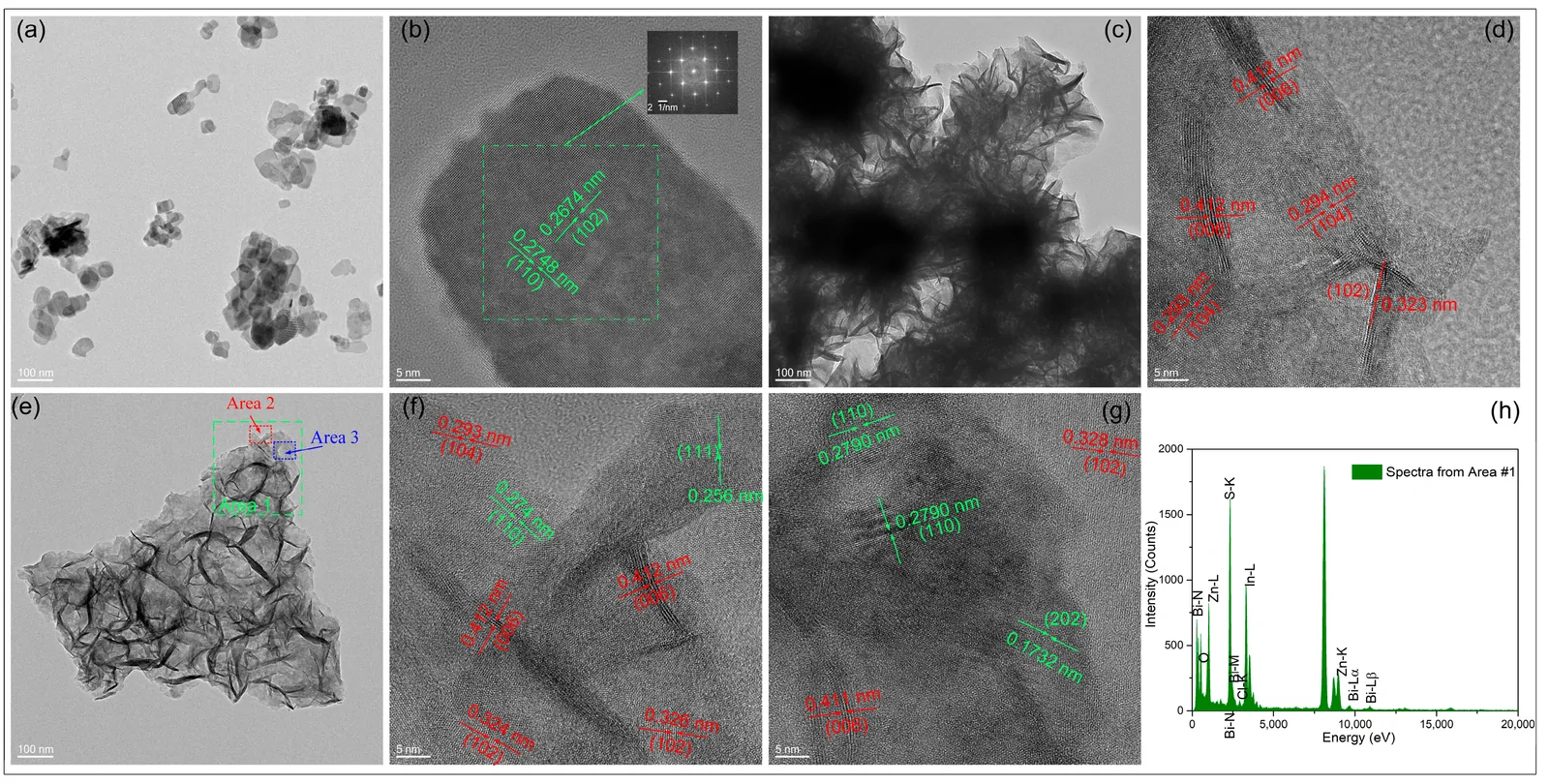
(**a**) TME image of BiOCl, (**b**) HRTME image of BiOCl, (**c**) TME image of ZIS, (**d**) HRTME image of ZIS, (**e**) TEM image of BiOCl@ZIS-25 heterojunction, (**f**,**g**) HRTEM image corresponding to area 2 and 3 in panel (**e**), (**h**) EDX spectrum of BiOCl@ZIS-25 heterojunction. The inset in panel (**b**) shows the fast Fourier transform pattern.

Figure 5 presents the TEM morphologies of BiOCl, ZIS, and the BiOCl@ZIS-25 composite. Pure BiOCl (Figure 5a) shows aggregated nanosheets, and HRTEM (Figure 5b) confirms lattice fringes of 0.2748 nm and 0.2674 nm, corresponding to the (110) and (102) planes of tetragonal BiOCl, as shown by the green labels/text in the HRTEM image. Moreover, the fast Fourier transform pattern obtained from this region (inset in Figure 5b) further confirms that the BiOCl nanosheets possess a single-crystal structure with good crystallinity. The TEM image of pure ZIS (Figure 5c) also shows significant agglomeration. The HRTEM image (Figure 5d) displays lattice fringes with spacings of 0.412 nm, 0.323 nm, and 0.293 nm (as shown by the red labels/text in the HRTEM image), which correspond to the (006), (102), and (104) planes of hexagonal wurtzite ZIS, consistent with the XRD results. In the BiOCl@ZIS-25 heterojunction (Figure 5e), ZIS grows along the 2D templates of BiOCl, reducing the overall agglomeration. This trend is more clearly observed in the supporting material (Figure S2 in Supporting Information). HRTEM (Figure 5f,g) shows the coexistence of ZIS, BiOCl, and Bi_2_S_3_ lattice fringes. EDS analysis of area 1 in Figure 5e confirms that the heterojunction is composed of Bi, Cl, O, Zn, In, and S elements, as shown in Figure 5h. Comprehensive analysis suggests that the addition of TAA preferentially sulfidizes the BiOCl surface to form a Bi_2_S_3_ interlayer, followed by the in situ growth of ZIS on the Bi_2_S_3_ surface, thereby constructing a Bi_2_S_3_ transition layer between BiOCl and ZIS. Furthermore, the BiOCl@ZIS-25 heterojunction significantly reduces the agglomeration observed in the pure BiOCl and ZIS phases, which is beneficial for enhancing the photocatalytic performance of the material.

**Figure 6:**
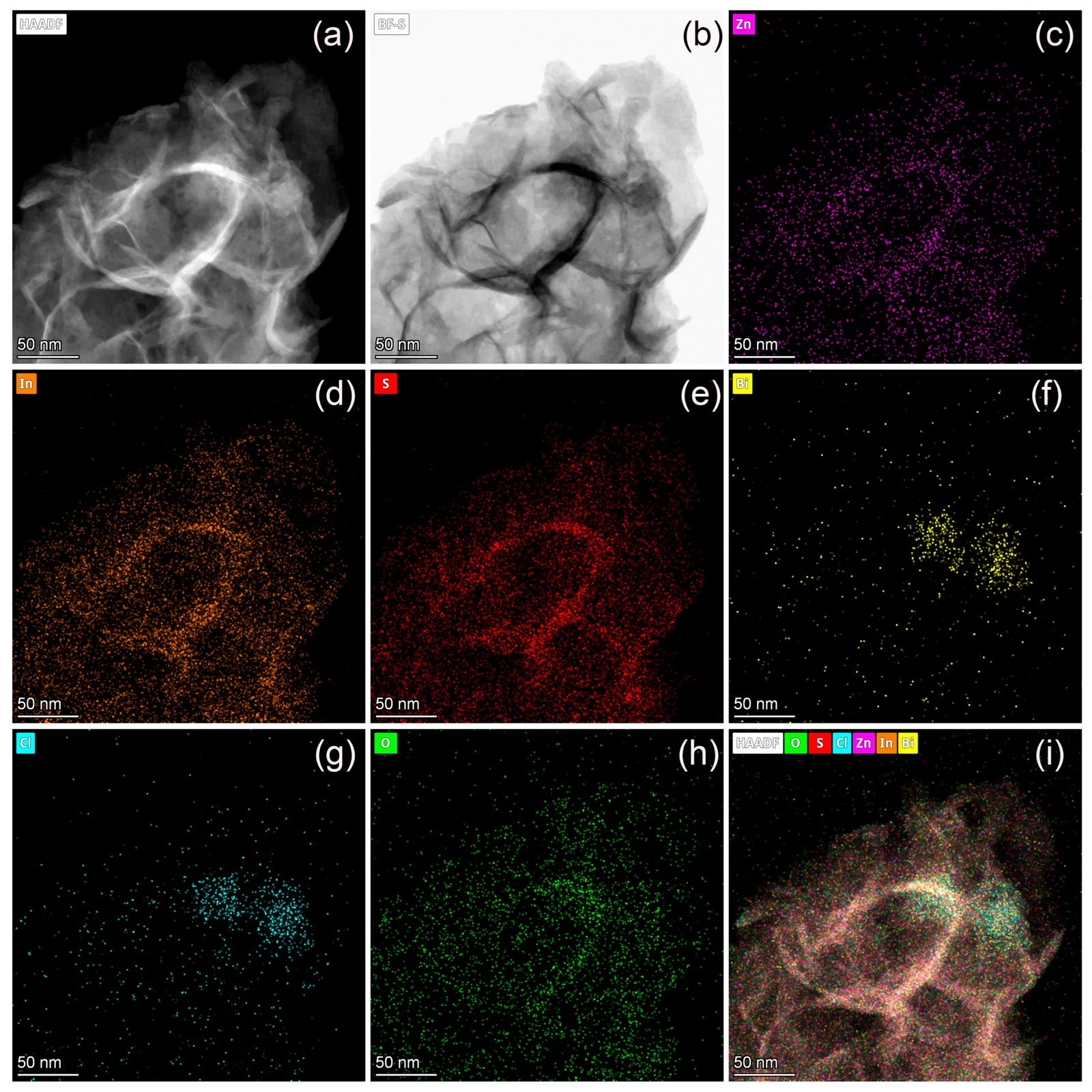
(**a**) High-angle annular dark-field STEM image, (**b**) bright-field STEM image, and (**c**–**i**) corresponding EDS elemental mapping of the BiOCl@ZIS-25 heterojunction.

Figure 6 shows the elemental distribution in the BiOCl@ZIS-25 heterojunction obtained through EDS mapping analysis. Figure 6a and Figure 6b present the high-angle annular dark-field image and the bright-field scanning transmission electron microscopy image of the corresponding region, respectively. The EDS elemental maps reveal that nanosheets composed of Bi, Cl, and O are attached to the surface of the ZIS nanosheets, indicating the successful formation of a heterojunction between BiOCl and ZIS.

**Figure 7:**
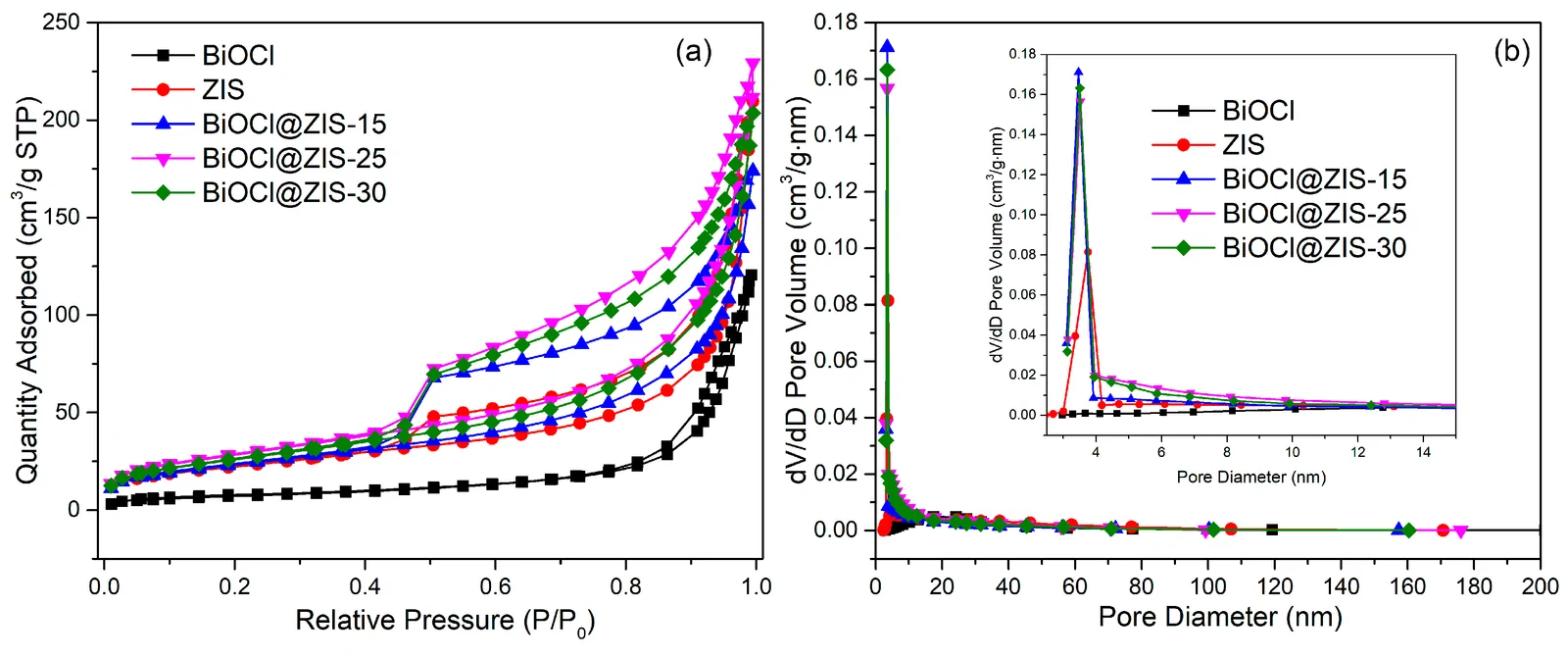
(**a**) Nitrogen adsorption–desorption isotherms and (**b**) pore size distribution curves of BiOCl, ZIS, and BiOCl@ZIS heterojunction.

As shown in Figure 7, all samples exhibit type IV isotherms with H3-type hysteresis loops, indicative of mesoporous structures formed by nanoparticle stacking [44,52]. As summarized in Table 1, the BiOCl@ZIS heterojunctions exhibit higher specific surface areas compared to pure-phase BiOCl and ZIS. Among them, the BiOCl@ZIS-25 heterojunction possesses the highest specific surface area (103.5133 m^2^·g^−1^), which is significantly larger than that of pure BiOCl (28.6515 m^2^·g^−1^) and ZIS (83.0818 m^2^·g^−1^). Concurrently, the pore volume of this heterojunction (0.356741 cm^3^·g^−1^) also exceeds that of pure BiOCl (0.186604 cm^3^·g^−1^) and ZIS (0.326241 cm^3^·g^−1^). Such structural features not only enhance the adsorption capacity of the catalyst toward organic pollutants but also provide more accessible active sites, thereby expanding the pathways for photocatalytic reactions and ultimately improving the photocatalytic performance of the material.

**Figure 8:**
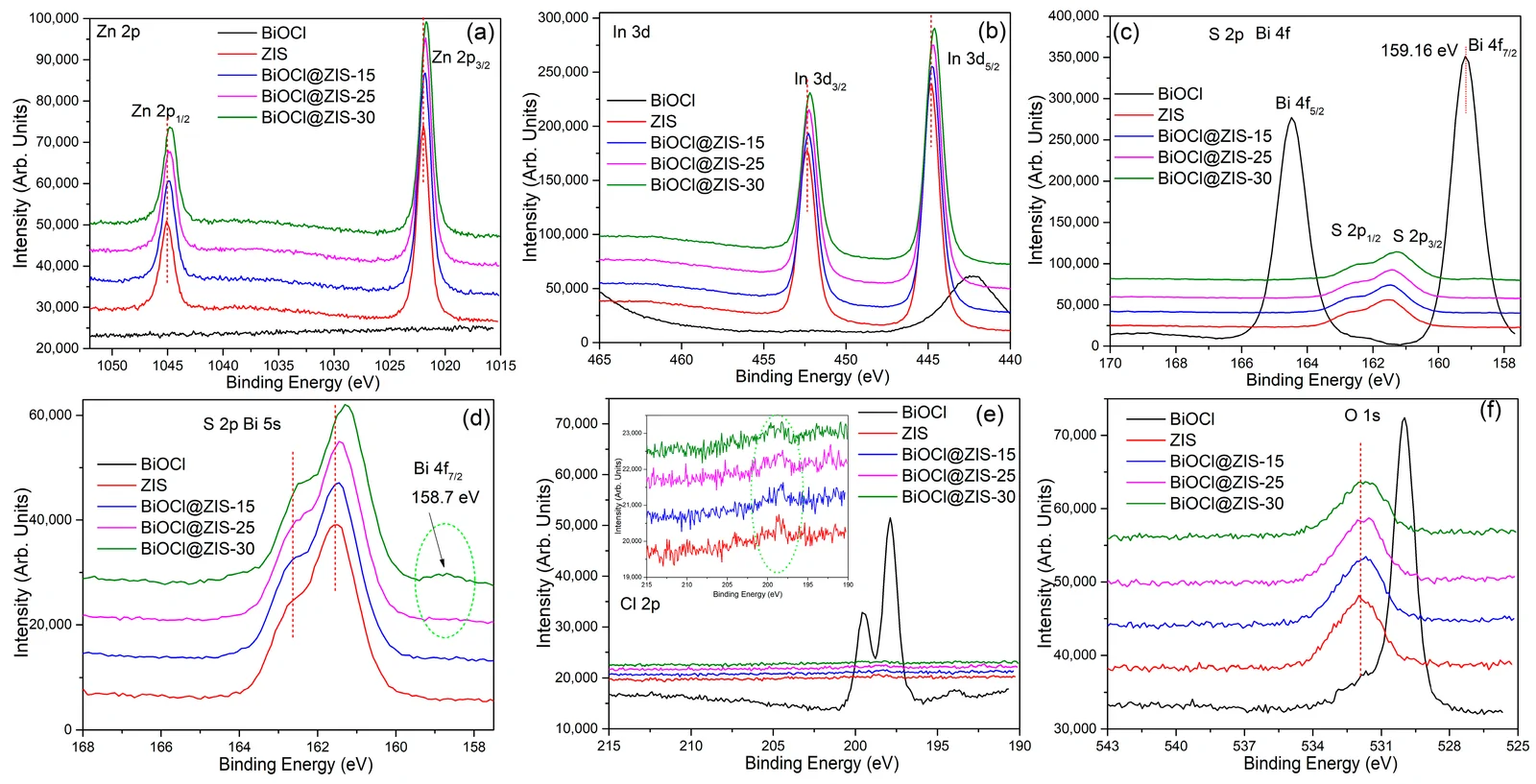
High-resolution XPS spectrum of BiOCl, ZIS, and BiOCl@ZIS heterojunction: (**a**) Zn 2p, (**b**) In 3d, (**c**) S 2p/Bi 4f, (**d**) enlarged view of panel (**c**), (**e**) Cl 2p, and (**f**) O 1s.

The surface chemical composition, elemental valence states, and interfacial interactions of BiOCl, ZIS, and the BiOCl@ZIS heterojunction were analyzed using XPS. The full-scan XPS spectra of the samples are provided in Supporting Information Figure S2. Since ZIS was grown in situ on the surface of BiOCl nanosheets via an oil-bath method, the composite surface is primarily covered by ZIS. As XPS is a surface-sensitive technique, the binding energy peaks of the BiOCl@ZIS heterojunction are largely consistent with those of pure ZIS. High-resolution XPS scans of Zn, In, S, Bi, Cl, and O are shown in Figure 8. All samples adsorb adventitious carbon when exposed to air, as extensively documented in the literature and evidenced by the C=C vibrational modes in FTIR spectra. Consequently, carbon signals were detected in the XPS survey spectra of all samples (Figure S3, Supporting Information). Thus, the C 1s peak at 284.5 eV served as a calibration reference for the binding energies of other elements. For pure BiOCl, the binding energy peaks located at 164.5 eV and 159.2 eV correspond to Bi 4f5/2 and Bi 4f7/2, respectively, which are assigned to the Bi^3+^ state [53,54]. The Cl 2p spectrum exhibits two peaks near 199.6 eV and 197.9 eV, attributed to the Cl^−^ 2p1/2 and 2p3/2 orbitals [43]. The O 1s spectrum can be deconvoluted into two peaks at 531.7 eV and 530.0 eV, corresponding to the Bi–O bond and surface hydroxyl groups (Bi–OH), respectively [55]. These characteristic binding energies of Bi 4f, Cl 2p, and O 1s confirm the successful preparation of high-purity BiOCl. For pure ZIS, the Zn 2p spectrum shows two peaks at 1022.0 eV and 1045.1 eV, assigned to Zn^2+^ 2p3/2 and 2p1/2 orbitals [26]. The In 3d spectrum displays peaks at 452.3 eV and 448.3 eV, corresponding to In 3d3/2 and In 3d5/2 [27]. The S 2p spectrum exhibits peaks at 161.6 eV and 162.7 eV, which can be ascribed to S 2p3/2 and S 2p1/2 [28]. After the formation of the ZIS/BiOCl heterojunction, as shown in Figure 8a,b,d, the binding energies of Zn 2p, In 3d, and S 2p shift toward lower values with increasing BiOCl content. This indicates a strong interfacial interaction (e.g., van der Waals forces) between ZIS and BiOCl, which favors the separation of photogenerated carriers and thus enhances the photocatalytic activity [43,49]. The negative shift in binding energy generally reflects an increase in electron density [56], suggesting that ZIS receives electrons from the intermediate Bi_2_S_3_, which is crucial for understanding the photocatalytic degradation mechanism. Furthermore, from the enlarged view of the S 2p (Bi 4f) region (Figure 8d) and the inset of Figure 8e, weak Bi 4f7/2 and Cl 2p signals are observed in the BiOCl@ZIS-30 sample, confirming the presence of BiOCl in the heterojunction. This result is consistent with the previous XRD and HRTEM analyses. To determine the band structure and carrier transport path of the heterojunction, valence-band XPS (VB-XPS) was directly performed on BiOCl and ZIS (Supporting Information Figure S4). The measured valence-band potentials (E_VB,XPS_) of BiOCl and ZIS are 3.99 eV and 1.41 eV, respectively. Using the formula E_VB,NHE_ = φ + E_VB,XPS_ − 4.44 (eV), (where φ is the instrument work function, 4.26 eV) [46,57], the valence-band potentials versus the normal hydrogen electrode (NHE) were calculated to be 3.81 eV for BiOCl and 1.23 eV for ZIS. Combined with the optical bandgap values, the conduction-band potentials (E_CB,NHE_) were derived as 0.20 eV for BiOCl and –1.45 eV for ZIS.

**Figure 9:**
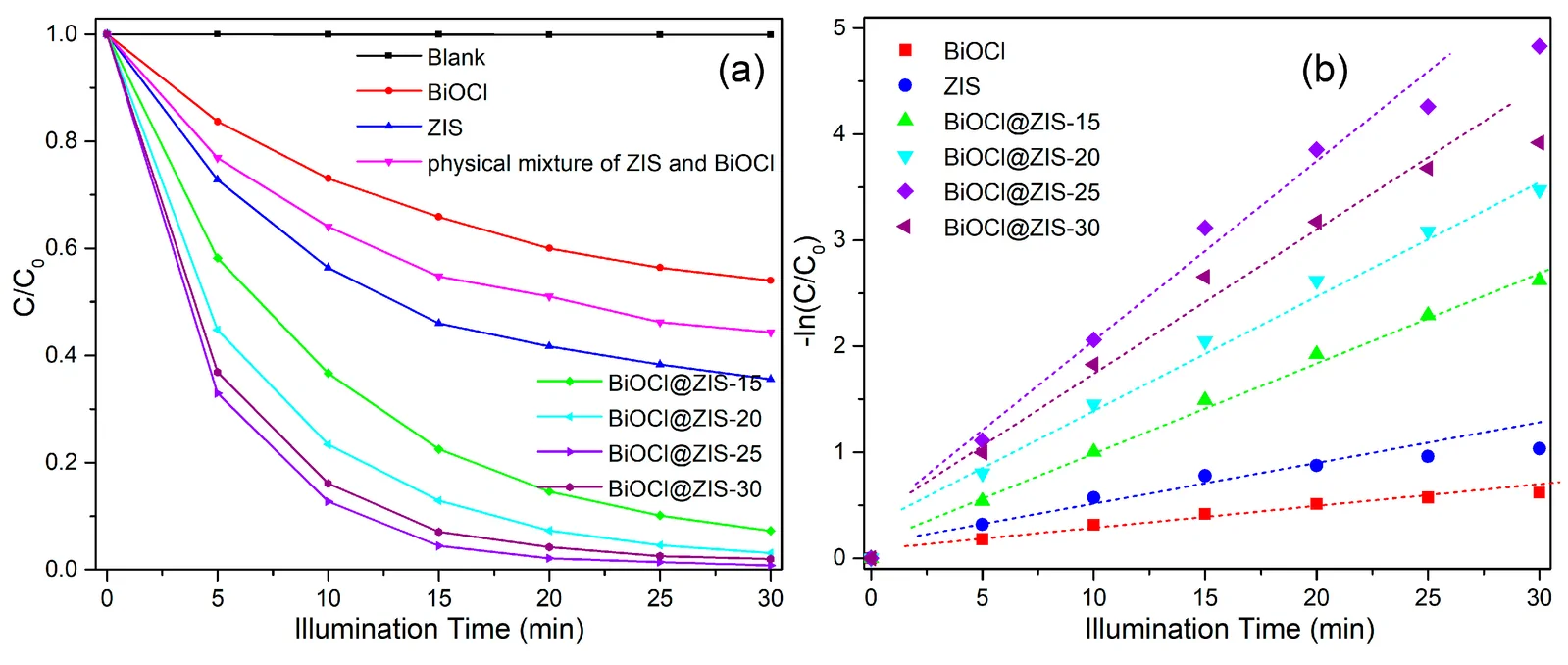
(**a**) Photocatalytic degradation of MB under visible-light irradiation for BiOCl, ZIS, BiOCl@ZIS heterojunction, and physical mixture of ZIS and BiOCl, and (**b**) corresponding pseudo-first-order kinetic curves for BiOCl, ZIS, and BiOCl@ZIS heterojunction.

**Figure 10:**
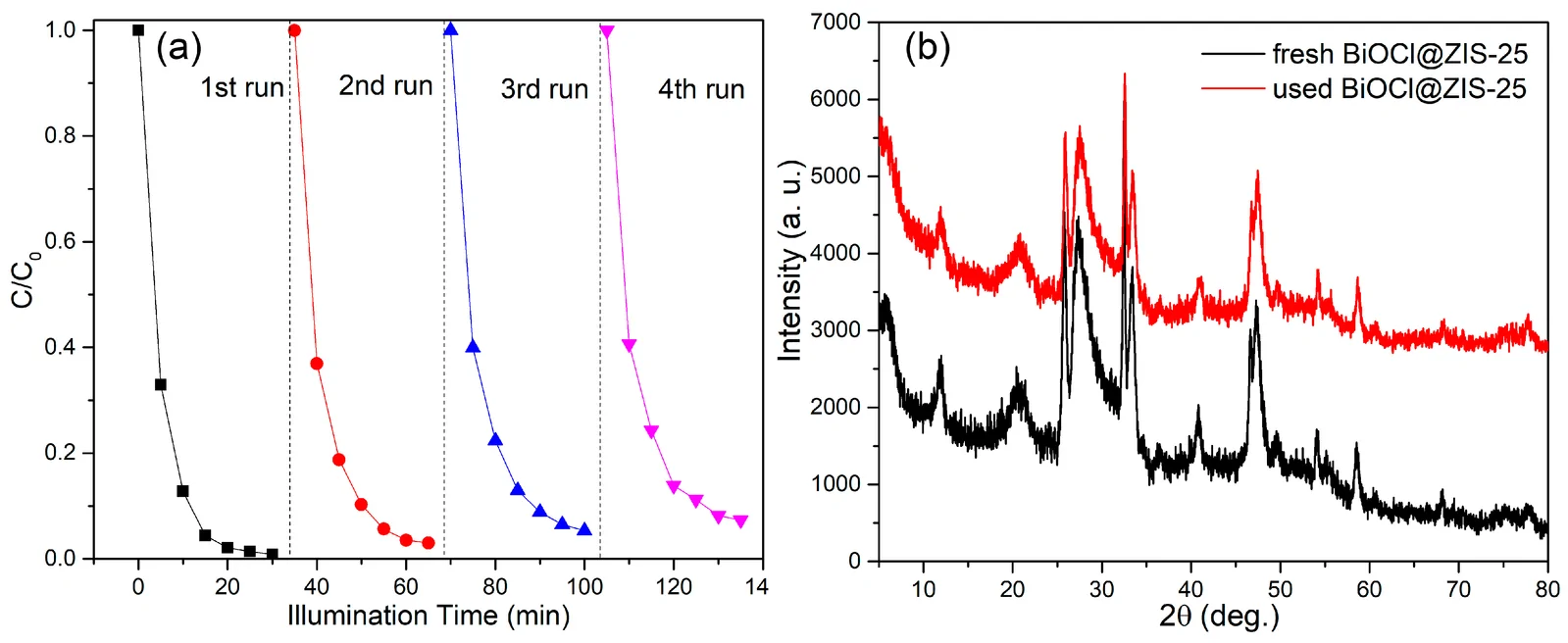
(**a**) Cycle performance of BiOCl@ZIS-25 to degrade MB, (**b**) XRD patterns of BiOCl@ZIS-25 before photocatalysis and after reuses.

The photocatalytic performance of BiOCl, ZIS, and the BiOCl@ZIS heterojunction was evaluated by degrading MB under visible-light irradiation. The degradation efficiency of the organic dye was calculated using the following formula: degradation efficiency (%) = (1 − C/C_0_) × 100%, where C_0_ is the initial MB concentration after adsorption equilibrium, and C is the residual concentration after the reaction [5]. To rule out any photolytic effect, a control experiment was performed by irradiating an MB solution in the absence of any catalyst. As shown in Figure 9a, light irradiation alone caused negligible degradation of MB. It was also observed that both pure BiOCl nanosheets and pure ZIS exhibited low photocatalytic efficiency, mainly owing to their limited visible-light absorption and rapid recombination of photogenerated charge carriers. In contrast, the BiOCl@ZIS heterojunction displayed significantly enhanced photodegradation activity. Among the heterojunction samples, BiOCl@ZIS-25 achieved the highest efficiency, with 97.88% MB degradation within 20 min. However, further increasing the BiOCl content (e.g., BiOCl@ZIS-30) led to a decline in performance, which could be attributed to excessive BiOCl compromising the dispersion of the heterojunction and reducing its effective interfacial contact area. To verify that the interfacial coupling between BiOCl and ZIS is the primary driver of the observed photocatalytic degradation, a physical mixture of ZIS and BiOCl was prepared and tested under identical conditions. As can be seen from Figure 9a, the physical mixture exhibited considerably lower degradation efficiency than the heterojunction. This result further confirms that the in situ-grown BiOCl@ZIS heterojunction plays a critical role in the photocatalytic degradation of MB. Meanwhile, to determine the degree of mineralization of MB, the total organic carbon (TOC) of the MB solution before and after reaction over the BiOCl@ZIS-25 catalyst was monitored during light irradiation, and the results are shown in Supporting Information Figure S5. The TOC results confirm that approximately 96.7% of the MB was mineralized within 30 min, indicating that the degradation of MB is predominantly driven by photodegradation rather than merely by adsorption of MB onto the catalyst. The kinetics of the MB degradation process can be described by a pseudo-first-order rate equation: −ln(C_0_/C) = kt [58], where k is the photodegradation rate constant. The fitting results are shown in Figure 9b. The results indicate that the photocatalytic reaction follows pseudo-first-order kinetics. The fitted rate constants k for BiOCl, ZIS, BiOCl@ZIS-15, BiOCl@ZIS-20, BiOCl@ZIS-25, and BiOCl@ZIS-30 are listed in Table 2. It can be observed that BiOCl@ZIS-25 exhibits the highest photocatalytic degradation rate, which is approximately 8 times and 4 times higher than that of pure BiOCl and pure ZIS, respectively. More importantly, as shown in Figure 10a, the degradation efficiency of MB over the BiOCl@ZIS-25 catalyst remained above 95% after four consecutive reuses, indicating that BiOCl@ZIS-25 exhibits excellent stability for the photocatalytic degradation of MB. The XRD patterns before and after use are shown in Figure 10b, where it can be observed that the phase of BiOCl@ZIS-25 remains largely unchanged, further confirming that BiOCl@ZIS has good reusability.

**Figure 11:**
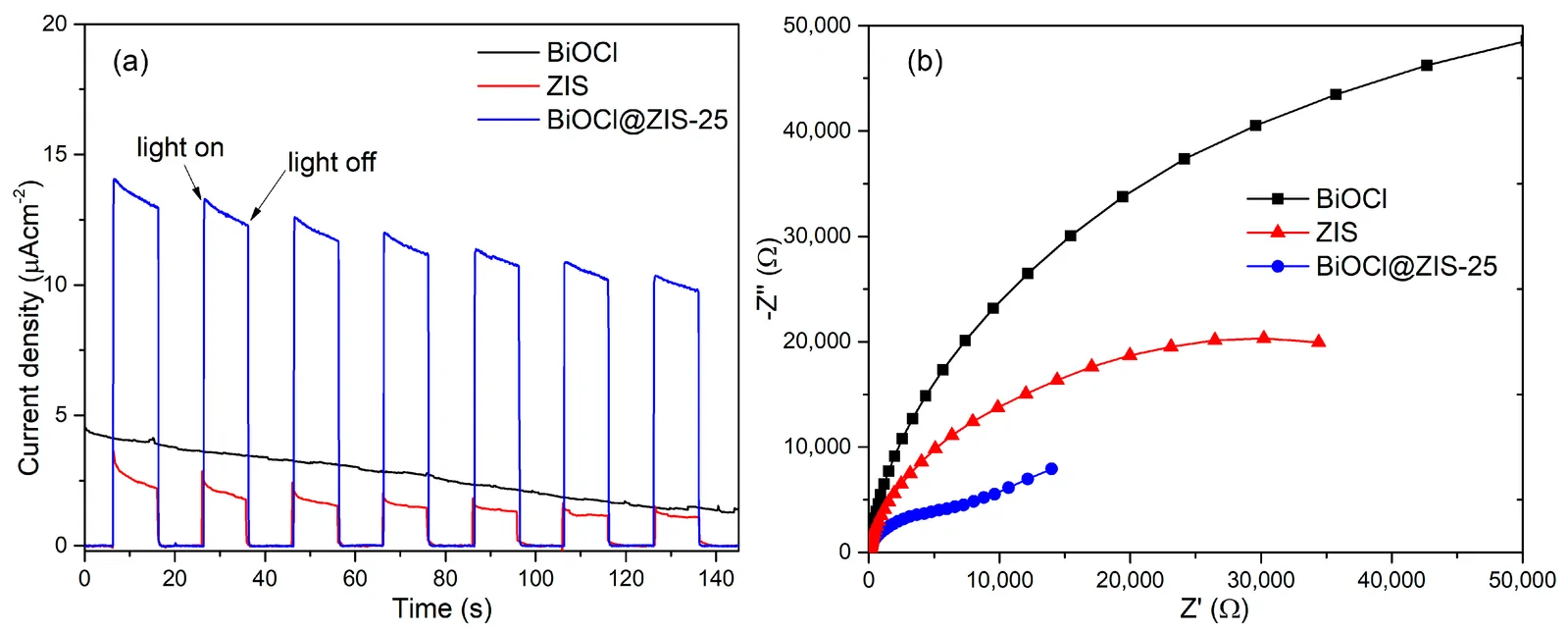
(**a**) Transient photocurrent responses, and (**b**) EIS Nyquist plots of BiOCl, ZIS, and BiOCl@ZIS-25 heterojunction.

To gain deeper insight into the physical mechanism behind the enhanced photocatalytic degradation performance, the samples were subjected to photocurrent response and EIS measurements, as shown in Figure 11a,b. The photocurrent response curves indicate that the BiOCl@ZIS-25 heterojunction exhibits the highest photocurrent intensity, demonstrating its superior ability for photogenerated carrier generation and separation. EIS spectra are commonly used to characterize the electron transfer resistance within heterojunction structures [37,38], thereby providing further evidence for the successful construction of the heterojunction (Figure 11b). A magnified view of the EIS spectra with the 1 Hz position marked is shown in Figure S6 of the Supporting Information. Generally, a smaller semicircle radius in the EIS curve corresponds to lower electron transfer resistance and faster charge transport. As observed in Figure 11b, the EIS semicircle radius of BiOCl@ZIS-25 is significantly smaller than those of pure BiOCl and ZIS, indicating its higher efficiency in separating photogenerated carriers.

**Figure 12:**
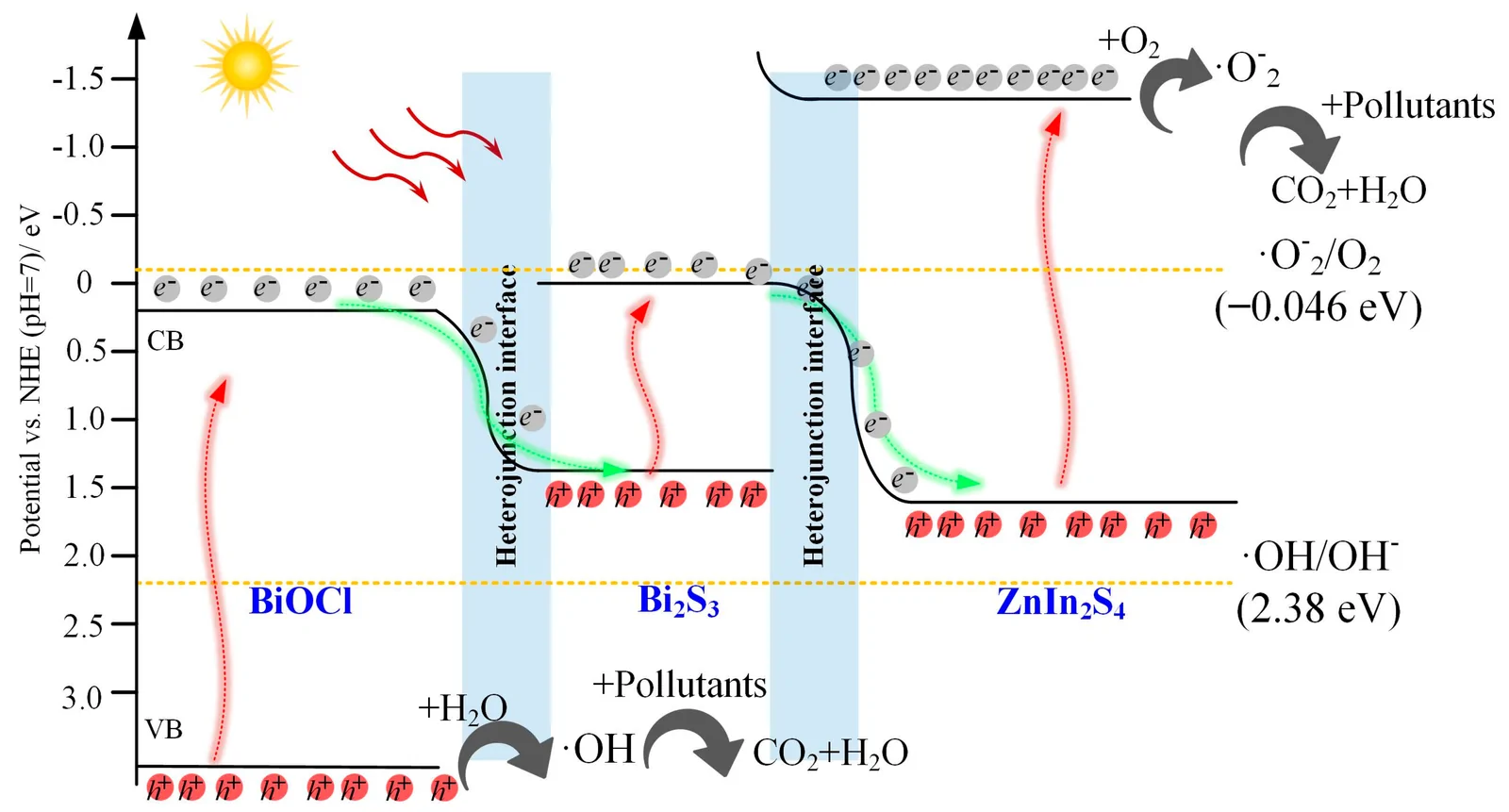
Schematic illustration of proposed photocatalytic mechanism of the enhanced performance of BiOCl@ZIS heterojunction.

Based on a comprehensive analysis of the microstructure and band structure of the catalyst, this work proposes a charge transfer mechanism for photogenerated electrons and holes during the photocatalytic degradation of organic dyes by the BiOCl@ZIS heterojunction, as illustrated in Figure 12. During the in situ growth of ZIS on the surface of BiOCl nanosheets, a Bi_2_S_3_ semiconductor forms at the interface, creating an additional heterojunction on top of the original one. Under simulated sunlight irradiation, electrons in the valence bands of BiOCl, Bi_2_S_3_, and ZIS are excited and jump to the conduction bands, generating photogenerated electrons and leaving photogenerated holes in the valence bands. According to the band alignment, electrons in the conduction band of BiOCl tend to recombine with holes in the valence band of Bi_2_S_3_, while electrons in the conduction band of Bi_2_S_3_ readily recombine with holes in the valence band of ZIS (this process is also supported by the low-energy shifts of Zn, In, and S binding energies observed in XPS). Through these recombination pathways, photogenerated holes in the valence band of BiOCl and photogenerated electrons in the conduction band of ZIS are more effectively separated. Subsequently, the separated electrons in the conduction band of ZIS can react with dissolved oxygen in water to form ·O_2_^−^ radicals, while the holes in the valence band of BiOCl react with water to produce ·OH radicals. These two active radicals play a key role in degrading organic pollutants, gradually mineralizing organic dyes into non-polluting water and carbon dioxide. Therefore, the photocatalytic process of the BiOCl@ZIS heterojunction follows a double Z-scheme charge transfer mechanism. Additionally, EPR spectroscopy further confirms the generation of radicals in the reaction system under light irradiation.

**Figure 13:**
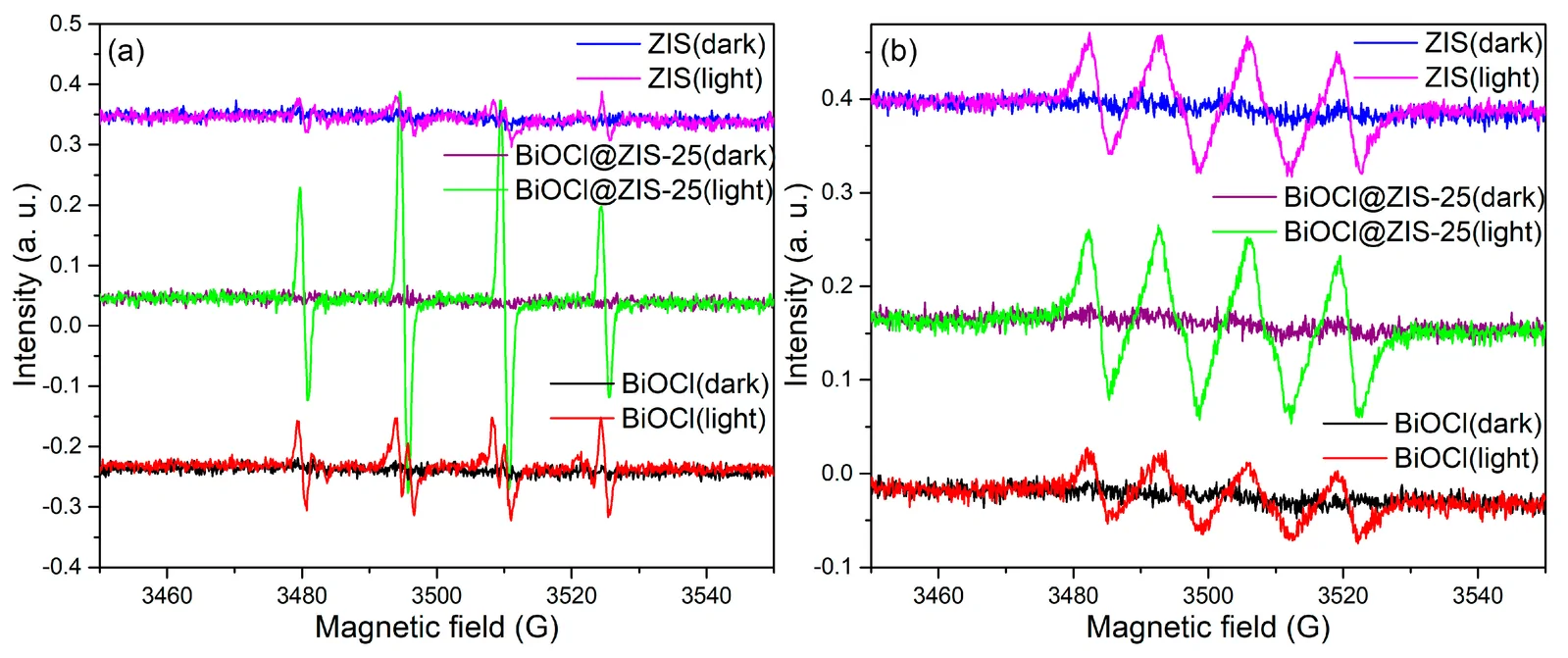
EPR spectra of (**a**) DMPO-OH and (**b**) DMPO-O_2_^−^ adducts for BiOCl, ZIS, and BiOCl@ZIS-25 heterojunction measured in the dark and under the full-spectrum irradiation from a 500 W Xenon lamp.

Figure 13 presents the EPR spectra obtained using DMPO as a spin-trapping agent to detect superoxide radicals (·O_2_^−^) and hydroxyl radicals (·OH) in solution. Under dark conditions, no significant radical signals are observed for BiOCl, ZIS, or the BiOCl@ZIS-25 heterojunction, indicating the absence of photogenerated electron-hole pairs without illumination. In contrast, upon irradiation with a xenon lamp, characteristic signals of ·OH and ·O_2_^−^ appear for all three samples, confirming the successful generation of these two active radicals during the photocatalytic process. Notably, the BiOCl@ZIS-25 heterojunction exhibits the highest EPR signal intensity, suggesting the greatest concentrations of ·OH and ·O_2_^−^ in its system. This result demonstrates that the BiOCl@ZIS heterojunction effectively promotes the separation of photogenerated electrons and holes. The separated carriers subsequently react with dissolved oxygen and water, respectively, producing more ·O_2_^−^ and ·OH, which in turn drive the efficient degradation of organic dyes through radical-based reactions. These findings provide additional support for the rationality of the double Z-scheme photocatalytic mechanism proposed earlier.

## 3. Experimental Procedure

### 3.1. Materials

All chemicals were of analytical grade and used as received. Bismuth nitrate pentahydrate (Bi_2_(NO_3_)_3_·5H_2_O, 99.99%), potassium chloride (KCl, 99.995%), polyvinylpyrrolidone (PVP, ~10,000), hydrochloric acid (HCl, 37%), glycerol (99.5%), zinc chloride (ZnCl_2_, 99.99%), indium chloride tetrahydrate (InCl_3_·4H_2_O, 99.99%), thioacetamide (TAA, 99%), and sodium sulfate (Na_2_SO_4_) were purchased from Aladdin Reagent Co. (Shanghai, China). Ethanol was of analytical grade. Deionized water (electrical conductivity: 18 MΩ·cm) was used throughout the experiments.

### 3.2. Synthesis of BiOCl Nanoplates

BiOCl nanoplates were synthesized using a straightforward hydrothermal method. Typically, for this procedure, 200 mg of PVP and 5 mmol of Bi_2_(NO_3_)_3_·5H_2_O were dissolved in 50 mL of deionized water under continuous stirring for 30 min. Subsequently, 4 mL of 37% hydrochloric acid was added to the solution, followed by another 30 min of stirring. Then, 10 mmol of KCl was introduced, and the mixture was stirred for an additional 30 min. The resulting solution was transferred into a 100 mL Teflon-lined stainless-steel autoclave and maintained at 180 °C for 12 h. After cooling naturally to room temperature, the product was collected and washed repeatedly with ethanol and deionized water to remove residual reactants. Finally, the purified sample was freeze-dried to obtain BiOCl nanoplates as a precursor for constructing heterojunctions.

### 3.3. Synthesis of BiOCl@ZIS Nanocomposites

A predetermined amount of BiOCl was dispersed in a mixed solution of 20% glycerol and 80 mL deionized water, and ultrasonicated for 1 h. Subsequently, 245 mg of anhydrous ZnCl_2_, 1056 mg of InCl_3_·4H_2_O, and 721 mg of TAA were added to the ultrasonically dispersed BiOCl solution, and the mixture was stirred for 30 min to ensure complete dissolution. The resulting solution was transferred to a breaker and heated in an oil bath at 80 °C for 2 h. Upon completion of the reaction, the product was collected via vacuum filtration, washed 2–3 times with deionized water and ethanol, and subsequently freeze-dried to obtain a powdered sample. By varying the mass of BiOCl (5, 10, 15, 20, 25, and 30 mg), the corresponding nanocomposites were designated as BiOCl@ZIS-5, BiOCl@ZIS-10, BiOCl@ZIS-15, BiOCl@ZIS-20, BiOCl@ZIS-25, and BiOCl@ZIS-30, respectively. For comparison, pure ZIS was synthesized under the same experimental conditions without the addition of BiOCl.

### 3.4. Characterization

The crystal structures of the as-prepared BiOCl, ZIS, and BiOCl@ZIS nanocomposites were analyzed by X-ray diffraction (XRD, Bruker D8 Advance diffractometer (Billerica, MA, USA), Cu Kα radiation, *λ* = 1.541 Å) at an operated voltage of 40 kV and a current of 40 mA. Fourier-transform infrared (FTIR) spectra were recorded in the range of 400–4000 cm^−1^ using a Nicolet iS20 spectrometer (Thermo Fisher Scientific, Waltham, MA, USA). Morphological features were observed via scanning electron microscopy (SEM, Zeiss SIGMA 360, Oberkochen, Germany). Detailed microstructures and elemental distribution were further investigated using transmission electron microscopy (TEM, 200 kV acceleration voltage, Talos F200X G2, Thermo Fisher Scientific, Hillsboro, OR, USA) equipped with an energy-dispersive spectrometer (EDS) for mapping. For TEM analysis, the powder sample were ultrasonically dispersed in ethanol for 15 min, drop-cast onto a copper grid, and dried prior to imaging. The surface elemental compositions and chemical states were determined by X-ray photoelectron spectroscopy (XPS) with Al Kα excitation (*hv* = 1486.6 eV). Ultraviolet-visible diffuse reflectance spectra were recorded on a UH4150 spectrophotometer (Hitachi, Tokyo, Japan), and the optical bandgap were derived from the corresponding *Tauc* plot. Electrochemical measurements were performed on a CHI660E electrochemical workstation (Shanghai Chenhua, Shanghai, China) using a standard three-electrode configuration with 0.5 M Na_2_SO_4_ as the electrolyte. A platinum foil (10 × 10 mm) served as the counter electrode, and a saturated Ag/AgCl electrode was used as the reference electrode. The PLS-FX300HU xenon lamp (PerfectLight, Beijing, China) was used as the light source, and its output optical power density is 300mW/cm^2^. And a mechanical shutter (10 s on/off) was used for transient photocurrent response measurements. No external bias of any kind is applied. The step size for data acquisition is set to 0.05 s. Electrochemical impedance spectroscopy (EIS) was conducted at open-circuit potential over a frequency range of 0.01 Hz to 100 kHz with an AC amplitude of 10 mV. The types and concentrations of radicals generated during irradiation were identified by electron paramagnetic resonance spectroscopy (EPR, Bruker EMXplus-6/1) using 5,5-dimethyl-1-pyridin-N-oxide (DMPO) as the spin-trapping agent in deionized water and methanol (for ·OH) and methanol (for ·O_2_^−^).

### 3.5. Evaluation of Photocatalytic Degradation Performance

The photocatalytic performance of the BiOCl@ZIS heterojunction was evaluated using a PerfectLight PCX50C Discover photocatalytic degradation system. Methylene blue (MB) served as the model pollutant, and a Vlight multi-channel LED panel (simulating the AM1.5G solar spectrum, PerfectLight, Beijing, China) was used as the irradiation source. In a typical procedure, 50 mg of the catalyst was ultrasonically dispersed in 50 mL of a 10 mg/L MB solution for 30 min. The resulting suspension was stirred in a dark for 60 min to establish adsorption–desorption equilibrium. Prior to illumination, a 3 mL aliquot was collected to determine the initial concentration after dark adsorption. Under light irradiation, 3 mL aliquots were withdrawn at 5 min intervals and centrifuged to remove the catalyst particles. The supernatant was analyzed using UV-vis spectrophotometry (UH4150, Hitachi, Tokyo, Japan) to monitor the residual dye concentration, from which the photocatalytic degradation efficiency was quantified.

## 4. Conclusions

In this work, a double Z-scheme BiOCl@Bi_2_S_3_@ZIS heterojunction photocatalyst was successfully fabricated through an in situ assembly strategy. Comprehensive characterization reveals that during the oil-bath process, TAA preferentially reacts with the surface of BiOCl nanosheets to form a Bi_2_S_3_ interlayer, onto which ZIS subsequently grows in situ, leading to a tightly coupled heterostructure. This architecture not only effectively suppresses nanoparticle aggregation but also significantly increases the specific surface area and the density of active sites. Optical and electrochemical measurements demonstrate that the heterojunction construction broadens the light-harvesting range, substantially improving the separation efficiency of photogenerated charges. Among the composites, BiOCl@ZIS-25 exhibits the highest photocatalytic activity, with a MB degradation efficiency under visible light that markedly surpasses those of the individual components. Radical-trapping experiments combined with band-structure analysis further clarify the double Z-scheme charge-transfer mechanism: Bi_2_S_3_ serves as an electron bridge that facilitates directional interfacial charge migration, enabling efficient separation of electrons in the conduction band of ZIS and holes in the valence band of BiOCl. This process yields abundant ·OH and ·O_2_^−^ active radicals, thereby driving the efficient mineralization of organic dyes. The present study offers a viable synthetic route and mechanistic understanding for developing highly efficient and stable bismuth-based/sulfide heterojunction photocatalysts.

## Figures and Tables

**Table 1:** BET specific surface area, pore volume and average pore size of BiOCl, ZIS, and BiOCl@ZIS heterojunction.

Sample	BET Surface Area (m^2^·g^−1^)	Pore Volume (cm^3^·g^−1^)	Pore Size (nm)
BiOCl	28.6515	0.186604	22.9165
ZIS	83.0818	0.326241	12.5924
BiOCl@ZIS-15	84.9724	0.270900	7.2240
BiOCl@ZIS-25	103.5133	0.356741	8.2361
BiOCl@ZIS-30	95.0881	0.316938	7.7865

**Table 2:** The rate constants (k) of BiOCl, ZIS, BiOCl@ZIS-15, BiOCl@ZIS-20, BiOCl@ZIS-25, and BiOCl@ZIS-30 heterojunction.

Sample	BiOCl	ZIS	BiOCl@ZIS-15	BiOCl@ZIS-20	BiOCl@ZIS-25	BiOCl@ZIS-30
The rate constants (min^−1^)	0.02074	0.03790	0.08393	0.10872	0.168616	0.13539

## Data Availability

The original contributions presented in this study are included in the article/Supplementary Material. Further inquiries can be directed to the corresponding author.

## Supplementary Materials

The following supporting information can be downloaded at https://www.mdpi.com/article/10.3390/molecules31162843/s1.
