# Supplementary material for: In Situ Fabrication of BiOCl@Bi2S3@ZnIn2S4 Double Z-Scheme Heterojunctions for Enhanced Photocatalytic Degradation Performance
Source: Molecules. 2026 Aug 14;31(16):2843. doi: 10.3390/molecules31162843 (PMC13515380; doi:10.3390/molecules31162843)
Supplement: Supplementary file 1 [file molecules-31-02843-s001.zip › molecules-4456018-supplementary.pdf]

Supporting Information for
In situ fabrication of $BiOCl@Bi_2S_3@ZnIn_2S_4$ double Z-scheme heterojunctions for enhanced photocatalytic degradation performance

Ligang Ma [1], Tingting Chen [1], Jingxuan Zhou [1], Jiulei Zhao [1], Xinlan Li [1], Huilin Jiang [1], Liping Li [1] and Xiaoqian Ai [2,*]

[1]School of Electronic Engineering, Nanjing Xiaozhuang University, Nanjing 211171, China

[2]School of Physics and Electronic Information, Jiangsu Second Normal University, Nanjing, 210013 China

[*]Corresponding author. E-mail address: aixiaoqian186@jssnu.edu.cn (Xiaoqian Ai)

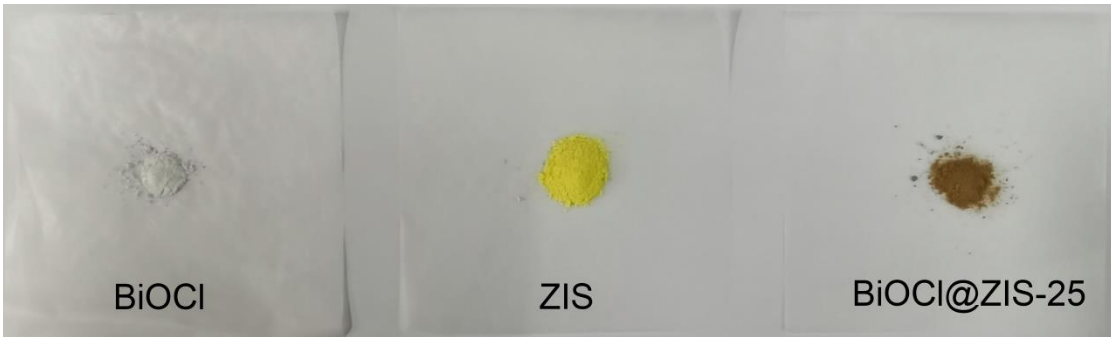

Figure S1. The photographs of the BiOCl, ZIS and BiOCl@ZIS-25 heterojunctions.

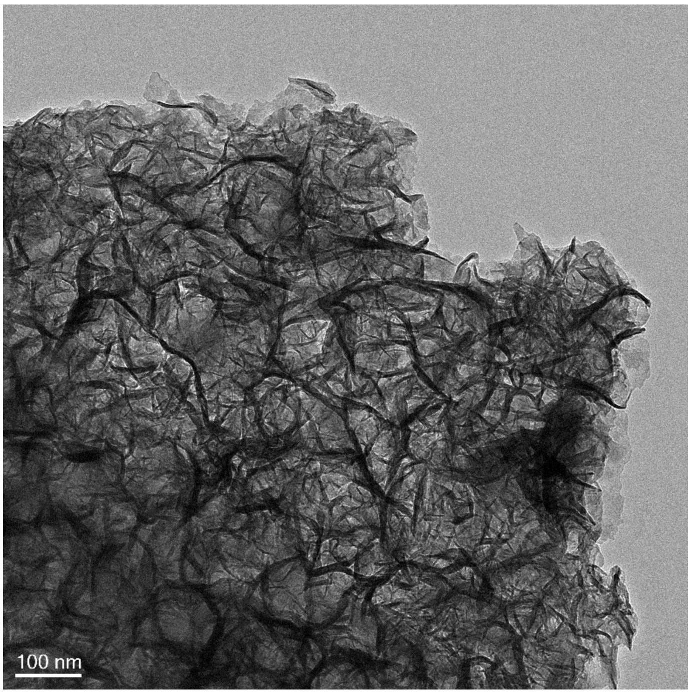

Figure S2. TEM image showing the morphology of the BiOCl@ZIS-25 heterojunction captured from another region.

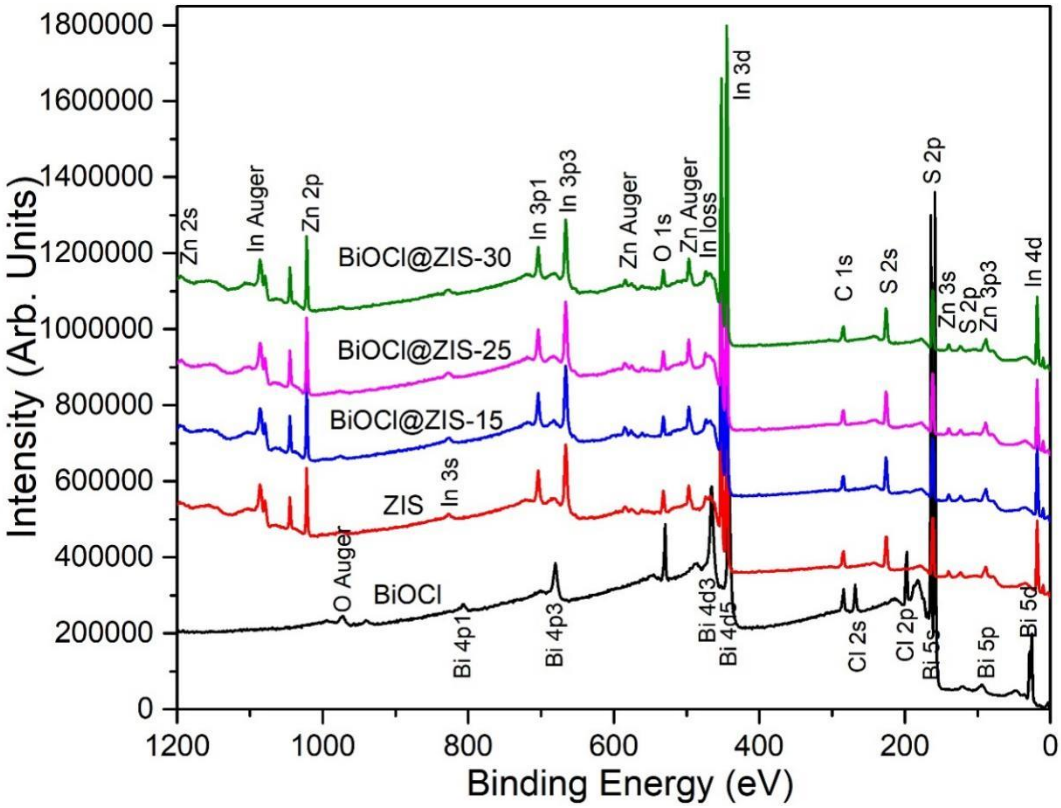

Figure S3. Full-spectrum XPS of BiOCl, ZIS, and BiOCl@ZIS heterojunction.

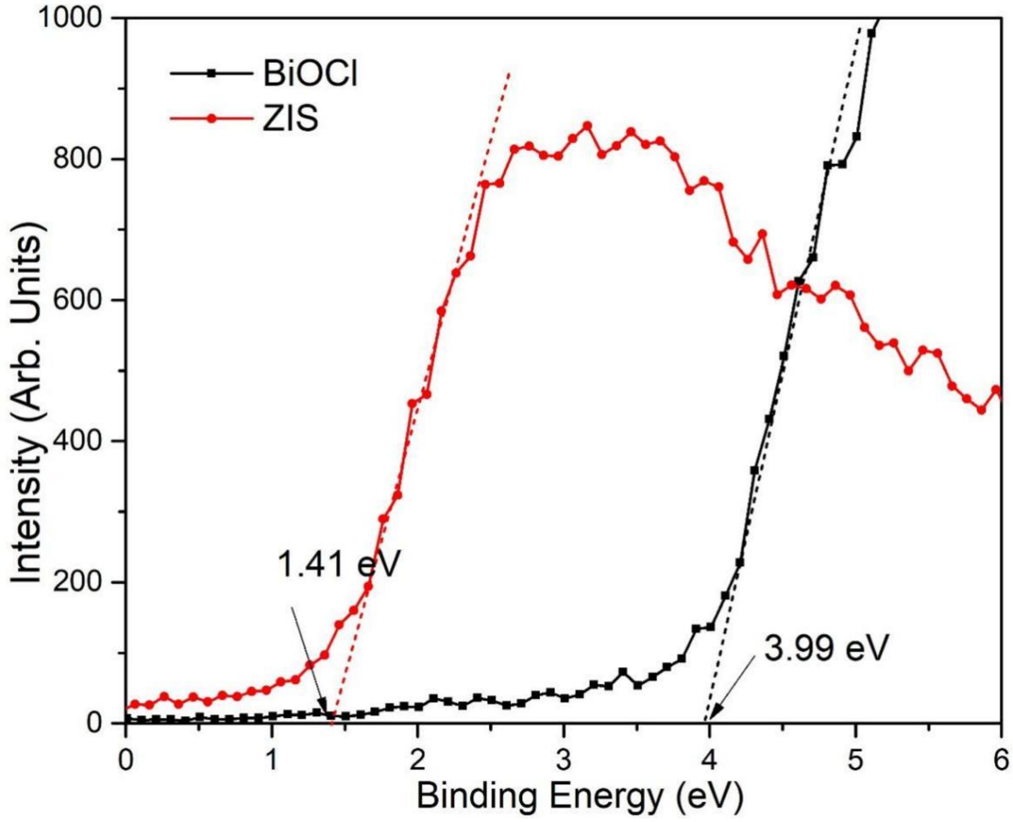

Figure S4. The band levels of XPS for BiOCl and ZIS.

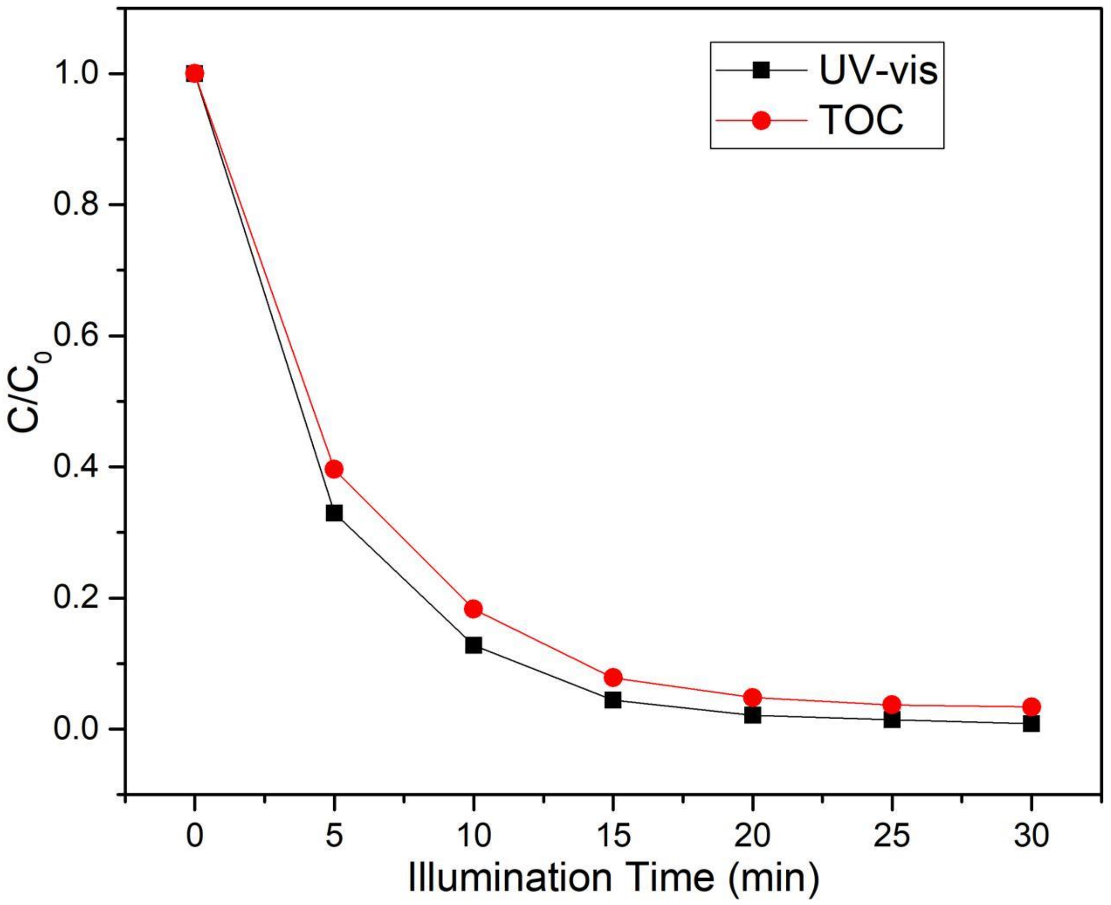

Figure S5. Photodegradation of MB catalyzed by BiOCl@ZIS-25 with respect to irradiation time under Uv-vis and TOC.

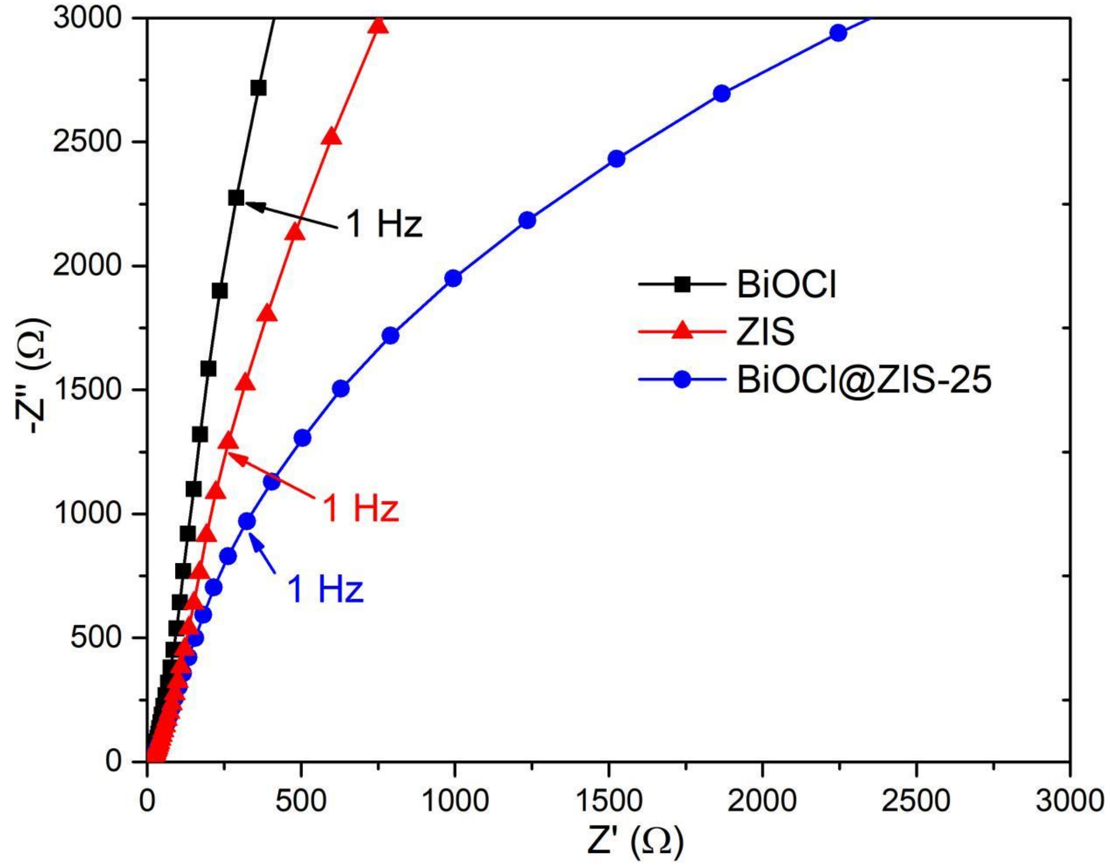

Figure S6. Magnified EIS plot with the 1 Hz position marked.
